# The Association Between the Mediterranean Diet and Fatty Acids in Red Blood Cells of Spanish Adolescents

**DOI:** 10.3390/nu17172888

**Published:** 2025-09-06

**Authors:** Nicolas Ayala-Aldana, David Lafuente, Iolanda Lázaro, Ariadna Pinar-Martí, Alexios Manidis, Sara Bernardo-Castro, Silvia Fernandez-Barres, Darren R. Healy, Martine Vrijheid, Oren Contreras-Rodríguez, Aleix Sala-Vila, Jordi Julvez

**Affiliations:** 1 Clinical and Epidemiological Neuroscience (NeuroÈpia), Institut d’Investigació Sanitària Pere Virgili (IISPV), 43204 Reus, Spain; nicolas.ayala@iispv.cat (N.A.-A.); ariadna.pinar@iispv.cat (A.P.-M.); alexios.manidis@iispv.cat (A.M.); sara.bernardo@iispv.cat (S.B.-C.); 2 Barcelona Institute for Global Health (ISGlobal), 08003 Barcelona, Spain; martine.vrijheid@isglobal.org; 3 Faculty of Medicine and Health Science, Hospital Clinic, University of Barcelona, 08036 Barcelona, Spain; 4 Facultat d’Enologia, Universitat Rovira i Virgili, 43007 Tarragona, Spain; davidlafuperezz@gmail.com; 5 Cardiovascular Risk and Nutrition, Hospital del Mar, Medical Research Institute, 08003 Barcelona, Spain; ilazaro@researchmar.net (I.L.); asala3@imim.es (A.S.-V.); 6 Centro de Investigación Biomédica en Red de la Fisiopatología de la Obesidad y Nutrición (CIBEROBN), Instituto de Salud Carlos III, 28029 Madrid, Spain; 7 Departament de Ciències Experimentals i de la Salut, Facultat de Medicina i Ciències de la Vida, Universitat Pompeu Fabra (UPF), 08002 Barcelona, Spain; 8 Agència de Salut Pública de Barcelona, Pl. Lesseps 1, 08023 Barcelona, Spain; silviabarres@gmail.com; 9 Institute of Public Health and Clinical Nutrition, Faculty of Health Sciences, School of Medicine, University of Eastern Finland, 70211 Kuopio, Finland; darren.healy@uef.fi; 10 Unitat Mixta de Neurociència Traslacional, Hospital Universitari Parc Taulí, INc-UAB, 08208 Barcelona, Spain; oren.contreras@uab.cat; 11 Department of Psychiatry and Forensic Medicine, Autonomous University of Barcelona, 08193 Barcelona, Spain; 12 Centro de Investigación Biomédica en Red Salud Mental (CIBERSAM), Instituto de Salud Carlos III, 28029 Madrid, Spain; 13 The Fatty Acid Research Institute, Sioux Falls, SD 57106, USA; 14 CIBER Epidemiología y Salud Pública, Instituto de Salud Carlos III, 28029 Madrid, Spain; 15 Human Nutrition Unit, Facultat de Medicina i Ciències de la Salut, Universitat Rovira i Virgili, 43201 Reus, Spain

**Keywords:** Mediterranean diet, fatty acids, polyunsaturated fatty acids, adolescence, principal component analysis

## Abstract

**Objective:** The Mediterranean diet (MedDiet) is characterized by its emphasis on plant-based foods, olive oil, and fish products, and has been associated with providing relevant fatty acids (FAs) for adolescent physiology. This study aims to investigate the relationship between adherence to the MedDiet and the FA composition of red blood cell (RBC) membranes in an adolescent population. **Methods:** The current research examines the relationship between MedDiet adherence, assessed using the KIDMED questionnaire, and the composition of RBC membranes, specifically measuring 22 FAs in a cross-sectional analysis of adolescents from two cohorts (mean age = 14.55). Baseline data from 552 participants with complete dietary adherence and FA information were analyzed using multivariable regression models and principal component analysis (PCA) as confirmatory analysis. All regression models were adjusted by age, sex, body mass index, physical activity, maternal education and cohort enrollment. **Results:** Main results shown that “Good adherence” to the MedDiet was positively associated with omega-3 FAs, including eicosapentaenoic acid (β = 0.34; 95% CI: 0.17, 0.52; *p*-value < 0.001) and docosahexaenoic acid (β = 0.29; 95% CI: 0.11, 0.46; *p*-value = 0.001), and inversely associated with specific omega-6 FAs, such as arachidonic acid (β = −0.28; 95% CI: −0.46, −0.11; *p*-value = 0.002) and adrenic acid (β = −0.19; 95% CI: −0.30, −0.08; *p*-value < 0.001). PCA identified distinct FA patterns, with “Good adherence” to the MedDiet being associated with an increase in the omega-3 FAs pattern (β = 0.32; 95% CI: 0.14, 0.49; *p*-value < 0.001). These findings remained robust after multiple test comparisons. **Conclusions:** This study underscores the potential of the MedDiet to promote optimal RBC FA composition in healthy adolescents, characterized by high levels of omega-3 FAs and reduced levels of arachidonic acid and adrenic acid in RBC membranes.

## 1. Introduction

Adolescent development can be affected by cardiovascular diseases [1] and mental health disorders [2], with diet playing a crucial role in their progression [3]. Notably, the Mediterranean diet (MedDiet) has demonstrated a protective effect against these conditions during adolescence [4,5]. The MedDiet emphasizes plant-based foods, with olive oil as the primary fat source [6]. It includes moderate amounts of dairy, fish, and poultry, while eggs are consumed weekly and red meat sparingly [7,8].

The MedDiet provides essential fatty acids (FAs), characterized by low levels of saturated fatty acids (SFAs) and high levels of polyunsaturated fatty acids (PUFAs), particularly omega-3 FAs [9]. Omega-3 PUFAs, including alpha-linolenic acid (C18:3 *n*-3, ALA), eicosapentaenoic acid (20:5 *n*-3, EPA) and docosahexaenoic acid (22:6 *n*-3, DHA), play crucial roles in the structural, functional, and antioxidative processes of the cardiovascular system and adolescent brain [10]. These omega-3 species are obtained via dietary intake of fatty fish such as salmon, tuna and mackerel (EPA and DHA), as well as nuts, seeds, and their derivatives (ALA) [11]

The MedDiet has been associated with beneficial effects in adolescents, including a reduced risk of cardiovascular disease and milder depression symptoms, with lasting impacts into adulthood [12]. These effects may be partly explained by the MedDiet’s impact on the FA content in red blood cell (RBC) membranes [13,14]. RBCs play a crucial role in adolescent metabolism by delivering oxygen to the cardiovascular system and brain [15]. Maintaining membrane homeostasis is essential for their proper function, with FAs playing a key role in this process. There is growing interest in assessing FAs in RBCs due to their ability to provide an accurate reflection of dietary lipid intake [16,17]

Given that the MedDiet has been associated with better cardiovascular and mental health outcomes in adolescence, this dietary pattern may provide optimal levels of omega-3 FAs that are beneficial for overall adolescent health [18]. These beneficial effects may be mediated by omega-3 FAs in RBC membranes [13,14]. We aimed to investigate the association of adherence to the MedDiet and 22 RBC FAs through a cross-sectional and multi-cohort analysis of adolescents in Catalonia (Spain). We hypothesize that high adherence to the MedDiet during adolescence is positively associated with omega-3 FAs in RBC membranes.

## 2. Methods

The current study utilizes a cross-sectional design, drawing on baseline data from the Walnuts Smart Snack Dietary Intervention Trial (WSS) and the 14-year follow-up from the Spanish birth cohort of the Childhood and Environment (INMA) Project. The flowchart of WSS and INMA participants is presented in Supplementary Figure S1.

The WSS aimed to assess whether consuming 30 g of raw walnut kernels daily for six months could improve cognitive and socioemotional development in a group of healthy adolescents of Barcelona. The study involved 771 participants from 11 high schools, who were recruited over a year (2015–2016). Details of the clinical trial are outlined in the WSS protocol [19], which received approval from the Parc Salut Mar’s Clinical Research Ethics Committee (approval number: 2015/6026/I). In the current cross-sectional study, we analyzed the baseline data from WSS, focusing on a subsample of 14-year-olds with biological FA samples (*n* = 332).

The INMA study is a multi-centric study conducted across several Spanish cities. The project aims to investigate the impact of prenatal and postnatal environmental factors on fetal growth and childhood development, involving 3100 pregnant women and their children (2006–2008). Detailed information is available in the INMA protocol [20]. We based this study on the INMA-Sabadell cohort, which received permission from “Instituto Municipal de Asistencia Sanitaria” Research Ethic Committee (approval number: 2005/2106/I). Participants of the INMA project, which were enrolled in Sabadell (INMA-Sabadell, *n* = 657), have been assessed approximately every two years: at 6 and 14 months, and at 2, 4, 7, 9, 11, and 14 years of age. We focused on data from the 14th to 16th year of follow-up with biological FA determinations (*n* = 328).

### 2.1. Nutritional, Sociodemographic and Lifestyle Data

Nutritional, sociodemographic, clinical, and lifestyle data were collected through WSS baseline assessments and INMA-Sabadell follow up. Field technicians conducted in-person interviews with adolescent participants, while parents completed questionnaires at home, returning them through the school system in both studies.

Adherence to the MedDiet was assessed using the KIDMED questionnaire, which assigns a score based on intake of key food groups. These food groups include fruits, vegetables, legumes, seafood, cereals, nuts, dairy products, and olive oil. The 16-items KIDMED questionnaire was administered to the participants. Scores of 3 or below reflect poor adherence, 4 to 7 indicate average adherence, and scores of 8 or above signify good adherence [19,21]. Given the small number of participants with low adherence, the KIDMED index was dichotomized into two categories: “poor to average adherence” (scores 1–7) and “good adherence” (scores 8–12). Height (cm) and weight (kg) were measured following standard recommendations to compute the body mass index (BMI). BMI was computed using the reference population (z-score), and adjusting for age and sex according to the World Health Organization’s recommendations [22]. Physical activity and sociodemographic information (age, sex and maternal education) were collected by questionnaires. Physical activity can refer to any exercise or sports practiced out of school hours and was recorded in three categories: “sedentary to low physical activity”, “moderate”, and “active to quite active”. Age was recorded as a continuous variable, whereas sex (male/female) and maternal education (up to high school/university) were captured as dichotomous variables. “Maternal social class was recorded as a categorical variable: “High and upper-middle class”, “Middle class”, and “working class”. Cohort information (WSS/INMA-Sabadell) of participants was also collected.

### 2.2. Laboratory Protocol for Fatty Acids Analysis

Blood samples were collected from participants of both cohorts after an overnight fast. Subsequently, we subjected the samples to centrifugation at 2500× *g* for 20 min at 20 °C within 4 h of extraction. Packed RBCs were preserved at −80 °C until analysis of the FAs. The FA profile within the RBCs was determined using gas chromatography, coupled either to flame ionization detector (WSS) or to electron ionization mass spectrometry (INMA), as previously described [23,24]. In both cases, the measurement of each FA was expressed as a percentage of the twenty-two FAs identified in the sample (Supplementary Figure S2)

### 2.3. Principal Component Analysis

We performed Principal Component Analysis (PCA) on the FAs of adolescents from the WSS and INMA-Sabadell cohorts. This analysis included all FAs measured in both groups and those that were common to both cohorts. No variables or FAs were selectively chosen for convenience. PCA was utilized to address the high correlation among FAs in red blood cells (Supplementary Figure S3). Following the PCA, Principal Components (PCs) with eigenvalues greater than 2 were retained. A varimax rotation was then applied to these retained components to clarify the contribution of each variable within them, and loading scores were analyzed for each component. Components were described based on variable loadings exceeding |0.2|, with a detailed examination of variables with the highest loading scores. Standardized scores for each adolescent were calculated for each component to serve as “outcome variables” for subsequent regression analysis. Higher standardized scores reflect greater representation of participants in the PC with FAs that contribute positively to the component, while lower scores indicate greater representation of participants in the PC with FAs that contribute negatively to the component.

### 2.4. Statistical Analysis

Wilcoxon sum rank and Chi-square tests were employed to compare the KIDMED levels with sociodemographic characteristics and RBC FAs from both WSS and INMA-Sabadell participants. Additionally, associations between KIDMED, FAs, and FA PCs were evaluated using multivariable linear regression models. The KIDMED score was assessed as a dichotomous variable comparing “poor-to-average adherence” with “good adherence” to MedDiet (exposure), while FAs and PC standardized score were evaluated as continuous variables (outcomes). For all regression analyses, fully adjusted models were assessed with mandatory covariates, including age, biological sex, BMI z-score, physical activity, maternal education and cohort. Statistical analyses were exclusive to adolescents with complete information on the variables included in the models (n = 552). Missing data were not imputed.

For all regression models, a *p*-value below 0.05 was considered as statistically significant. We conducted the False Discovery Rate (FDR) test for multiple test comparisons on regression models. As the main criterion, we considered a threshold alpha value of 0.05 to calculate the q-values. After conducting FDR analyses, we considered exposure coefficients to be statistically significant when *p*-values were lower than q-values across all regression models.

Sex-stratified analyses were conducted to examine potential differences in associations by biological sex. Sensitivity analyses were performed to assess the influence of maternal social class and BMI on the observed relationships. Additionally, to evaluate potential cohort effects, interaction terms between MedDiet adherence (KIDMED categories) and cohort membership (KIDMED × cohort) were included in the models.

All analyses were conducted using R base (version 4.4.0) and R Studio (version 4.2.3). The “zscorer” library (version 0.3.1) was used to calculate BMI z-scores following World Organization Health’s recommendations [25], while the “factoextra” (version 1.0.7) and “psych” libraries (version 2.4.3) were utilized for performing PCA [26,27].

## 3. Results

The baseline characteristics of the study population are shown in Table 1. A large percentage of the mothers of participants with “good adherence” to MedDiet had a university-level education (60%), while only 45% of mothers in the “poor to average adherence” to MedDiet had university-level education. The majority of participants within the “good adherence” group to MedDiet engage in “active to quite active” physical activity (65%).

The baseline characteristics of RBC FAs are shown in Table 2. While the percentage differences in the contribution of different FAs to RBC membranes were relatively small between the “poor to average adherence” and “good adherence” groups to the MedDiet, many of these differences were statistically significant. Several omega-6 FAs, such as C18:3 *n*-6 (gamma-linolenic acid, GLA), C20:2 *n*-6 (eicosadienoic acid, EDA), C20:4 *n*-6 (arachidonic acid, AA), and C22:4 *n*-6 (adrenic acid, AdA) showed slightly higher levels in the “poor to average adherence” to MedDiet group (*p*-value < 0.001). Meanwhile, certain specific omega-3 FAs, such as C20:5 *n*-3 (EPA) (*p*-value = 0.004), and C22:6 *n*-3 (DHA) (*p*-value = 0.001), showed higher levels in the “good adherence” to MedDiet group.

We present only the statistically significant associations (*p*-value < 0.05) of adherence to the MedDiet (exposure) with RBC FAs (outcomes) in Table 3, while all multivariate regression outputs are provided in Supplementary Table S1. Adherence to the MedDiet, as measured by the KIDMED score, was associated with significant changes in RBC FAs in the adolescent participants. “Good adherence” to MedDiet group was positively associated with an increase in C16:0 (palmitic acid) (β = 0.18; 95% CI: 0.01, 0.35; *p* = 0.041) and all-trans C18:1 FAs (β = 0.05; 95% CI: 0.01, 0.09; *p* = 0.016) compared to the “poor-to-average” adherence to MedDiet. However, the “good adherence” group was inversely associated with C20:4 *n*-6 (AA) (β = −0.19; 95% CI: −0.30, −0.08; *p*-value < 0.001), C22:4 *n*-6 (AdA) (β = −0.28; 95% CI: −0.46, −0.11; *p*-value = 0.002), and C22:5 *n*-6 (docosapentaenoic omega-6, DPA omega-6) (β = −0.22; 95% CI: −0.40, −0.04; *p*-value = 0.018) compared to the reference group (“poor-to-average adherence” to MedDiet). Additionally, the “good adherence” to MedDiet group was positively associated with C20:5 *n*-3 (EPA) (β = 0.34; 95% CI: 0.17, 0.52; *p*-value < 0.001) and C22:6 *n*-3 (DHA) (β = 0.29; 95% CI: 0.11, 0.46; *p*-value = 0.001) compared to the “poor-to-average adherence” group.

**Figure 1:**
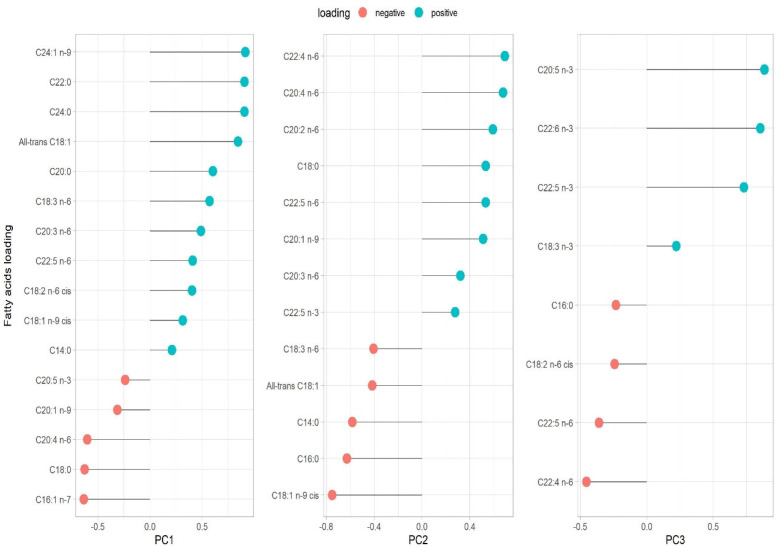
Loading variable for each principal component. Eigenvalue criteria >2.0. The variance (%) of each component is presented as follows: PC1 (33.75%), PC2 (13.22%), and PC3 (10.21%). The total variance explained, by these three principal components, is 57.20%. PC1 very-long chain FAs; PC2 long-chain omega-6 FAs, PC3 omega-3 FAs.

Three PCs were extracted using PCA; eigenvalue > 2.0 criteria for PC retention and loadings greater than |0.4| were considered statistically relevant for the pattern analysis (Supplementary Table S2). The three PCs retained accounted for 57.20% of the total variance of the FA data. No additional relevant information was added in the subsequent PCs, as confirmed by the scree plot (Supplementary Figure S4). Factor loadings for the FA patterns are presented as their loading contributions in Figure 1. PC1 was named “very-long chain FAs” as it was primarily characterized by, ordered from largest to smallest loading, C24:1 *n*-9 (nervonic acid), C22:0 (behenic acid), and C24:0 (lignoceric acid). Both C16:1 *n*-7 (palmitoleic acid) and C18:0 (stearic acid) showed negative loadings. PC2 was labelled “long-chain omega-6” because it was characterized by C20:4 *n*-6 (AA), C22:4 *n*-6 (AdA), and C20:2 *n*-6 (EDA, omega-6) as positive loadings. The FAs with negative loadings were C18:1 *n*-9 *cis* (oleic acid), C16:0 (palmitic acid), C14:0 (myristic acid) and all-trans C18:1. PC3 was named “omega-3 FAs” since it was mainly characterized by positive loadings of C20:5 *n*-3 (EPA), C22:6 *n*-3 (DHA), and C22:5 *n*-3 (docosapentaenoic acid, DPA), while the FA with negative loadings was C22:4 *n*-6 (AdA). The summary of the variables’ loadings for each component (PC1, PC2, and PC3) is also displayed in the PCA biplot (Supplementary Figure S5). Additionally, the correlation between each FA and the PC scores was also confirmed using Spearman correlation (Supplementary Table S3).

Adherence to the MedDiet, as measured by the KIDMED score, was associated with distinct changes in FA patterns (Table 4). “Good adherence” to the MedDiet group was inversely associated with long-chain omega-6 FAs PC (PC2) (β = −0.19; 95% CI: −0.36, −0.03; *p*-value = 0.020) compared to reference group (“poor-to-average adherence” to MedDiet). “Good adherence” to the MedDiet group was positively associated with the omega-3 FAs PC (PC3) (β = 0.32; 95% CI: 0.14, 0.49; *p*-value < 0.001). No significant associations of adherence to the MedDiet with PC1 (long-chain FAs pattern) were found.

FDR correction was applied to the significant associations of adherence to MedDiet with RBC FA composition (Supplementary Table S4). The following FAs remained statistically significant after FDR correction: C20:4 *n*-6 (*p*-value < 0.001; q-value = 0.004), C22:4 *n*-6 (*p*-value = 0.002, q-value = 0.010), C20:5 *n*-3 (*p*-value < 0.001; q-value = 0.002), C22:6 *n*-3 (*p*-value = 0.001; q-value = 0.008), and PC3 omega-3 FAs (*p*-value < 0.001; q-value = 0.006). These results indicate that the observed associations are unlikely to be due to multiple testing errors, reinforcing the robustness of the findings.

Our stratified analysis by sex revealed differential associations in the relationship between MedDiet adherence and fatty acid profiles (Supplementary Table S5). Significantly positive relationships were observed for long-chain omega-3 FAs in males compared to females (EPA: β = 0.46, 95% CI: 0.24, 0.68, *p*-value < 0.001; DHA: β = 0.32, 95% CI: 0.09, 0.54, *p*-value = 0.006; DPA: β = 0.26, 95% CI: 0.02, 0.49, *p*-value = 0.030). Conversely, inverse associations were found for specific omega-6 FAs in females (AA: β = −0.25, 95% CI: −0.42, −0.07, *p*-value = 0.005; AdA: β = −0.34, 95% CI: −0.61, −0.08, *p*-value = 0.012). Regarding sensitivity analyses, the addition of either BMI z-score or maternal social class to adjustment models resulted in coefficient changes of less than 20% for all FAs when comparing basic and fully adjusted models, indicating that these factors did not substantially influence the observed associations between MedDiet adherence and RBC FA concentrations (Supplementary Tables S6 and S7).

Finally, to evaluate potential cohort-related biases, we conducted an interaction analysis between MedDiet adherence and cohort (Supplementary Table S8). Statistically significant interaction effects (“Poor-to-average adherence” × “WSS cohort”) were observed for LA (β = 0.38, 95% CI: 0.03, 0.73, *p*-value = 0.032), DPA (β = −0.52, 95% CI: −0.86, −0.17, *p*-value = 0.004), DHA (β = −0.42, 95% CI: −0.78, −0.06, *p*-value = 0.022), and the omega-3 PC3 (β = −0.42, 95% CI: −0.78, −0.06, *p*-value = 0.021).

## 4. Discussion

In this cross-sectional study, we used data from adolescents in the WSS and INMA-Sabadell cohorts to analyze the associations of adherence to the MedDiet, as measured using the KIDMED score, with 22 FAs in RBC membranes. Our results show significant associations between the “good adherence” to the MedDiet group with FAs compared to the reference group (“poor-to-average adherence”) in fully adjusted models. Specifically, we observed inverse associations with omega-6 AA, and AdA, as well as positive associations with EPA, DHA, and the PC3 omega-3 FAs pattern. These findings remained consistent after correcting for multiple tests.

“Good adherence” to the MedDiet group was inversely associated with AA and AdA in RBCs. These inverse associations add to the growing evidence that individuals with good adherence to the MedDiet may exhibit lower levels of these omega-6 FAs in RBCs. A potential explanation for these findings lies in the dietary composition of the MedDiet, which emphasizes a high intake of omega-3 FA-rich foods, such as vegetables, fish, nuts, and seeds [28], while limiting the consumption of processed foods and vegetable oils high in omega-6 FAs [29]. At an epidemiological level, a cross-sectional study in children aged two to less than ten years old found an inverse association between total omega-6 FAs in whole blood with KIDMED scores [30]. Furthermore, an intervention promoting MedDiet adherence in adults with gingivitis, measured by FFQ before and after the intervention, showed decreased levels of LA, AA, and total omega-6 in the serum of the MedDiet intervention group [31]. Thus, our findings are consistent with previous research indicating that a good adherence to MedDiet is associated with lower levels of AA and AdA and replicates these results in healthy teenagers.

On the other hand, adherence to the MedDiet was positively associated with EPA and DHA. These findings are congruent with previous research showing that good adherence to MedDiet is associated with higher omega-3 composition in RBCs. EPA and DHA are predominantly found in fatty fish such as salmon, mackerel, sardines, and anchovies, as well as in fish oils and algae-based supplements [8,32]. These MedDiet food sources are important contributors to omega-3 FAs intake. Comparing our results with similar research, a report from HELENA study in European adolescents (*n* = 2330) showed a positive association between MedDiet score and omega-3 FAs in serum [33]. Other research using PCA-derived patterns found that a pattern characterized by the intake of fish, shrimp, crab, shellfish, leafy vegetables, nuts, and tubers was positively associated with omega-3 FAs and negatively associated with SFAs in a population of children aged 4 to 7 years with overweight or obesity [34]. In recent years, a systematic review on the relationship between MedDiet and omega-3 FAs was conducted which included 7 observational studies and 15 randomized controlled trials; all observational studies reported a positive relationship between adherence to the MedDiet and omega-3 PUFA tissue levels. Two-thirds of the randomized controlled trials showed significant increases in omega-3 FA concentrations [35].

Moreover, when focusing on highly correlated FAs, our findings revealed a positive association of adherence to the MedDiet with the PC3 omega-3 FAs pattern. This pattern was mainly enriched by C20:5 *n*-3 (EPA), C22:6 *n*-3 (DHA) and C22:5 *n*-3 (DPA). With a subtle differ-ence in the loading cut-off, 18:3 *n*-3 (ALA) is also present in the current PC. From a metabolic perspective, these FAs are interconnected through biosynthetic pathways. ALA serves as a precursor for the synthesis of longer-chain omega-3 FAs, including EPA, through a series of elongation and desaturation steps [36]. DPA, often considered an intermediary, can be converted to either EPA or DHA, depending on the metabolic demand and enzymatic activity [37]. The efficient metabolism of these PUFAs depends on factors such as dietary intake, genetic variation, and overall health, all of which can influence their relative abundance in the body [38,39]. In this manner, the association of adherence to MedDiet with the PC3 omega-3 pattern supports the idea that MedDiet foods intake is related to the percentage of related omega-3 FAs in RBC membranes. Furthermore, these PC3 omega-3 associations are consistent with the observed positive associations between adherence to the MedDiet and EPA and DHA in our previous regression models (Table 3).

Our sex-stratified analysis revealed notable differences in the associations between MedDiet adherence and FA profiles. In males, stronger positive relationships were observed for long-chain omega-3 FAs, including EPA, DHA, and DPA. In contrast, a more notable inverse relationship was observed in females for specific omega-6 fatty acids, including arachidonic acid (AA) and adrenic acid (AdA). Women may require higher amounts of omega-6, particularly AA, due to its crucial role in hormone synthesis. AA is an important precursor in the production of eicosanoids, compounds that regulate various physiological functions, including inflammatory responses and hormonal activity [40]. However, this is not observed in every case, underscoring the importance of considering sex-specific differences in fatty acid metabolism [41].

Moreover, maternal social class was included as a variable in the sensitivity analysis. No significant changes were observed in the coefficients of adherence to MedDiet exposure, either in the fully adjusted model or when additionally controlled for maternal social class. This demonstrates the robustness of variable selection in our model. However, it is important to note that previous studies analyzing the direct effect of social class on access to the MedDiet found that higher family income is associated with better adherence to this dietary pattern [42].

Finally, to evaluate potential cohort-related biases, we conducted an interaction analysis between MedDiet adherence and cohort. Significant interactions were observed for LA, DPA, DHA, and the omega-3 PC3, indicating that, relative to the reference group (“poor-to-average adherence”), the associations of good MedDiet adherence with these FAs differed between the WSS and INMA cohorts. These differences may reflect cohort-specific factors, including recruitment, socio-demographics, and RBC measurement protocols (flame ionization detector in WSS vs. electron ionization mass spectrometry in INMA). Despite this heterogeneity, the overall direction of associations remained consistent, supporting the robustness of our main findings.

Exploring the potential biological mechanisms, adolescence represents a period of increased metabolic demands where FA metabolism plays a central role. Adherence to the MedDiet may influence these processes through omega-3 FAs from fish, which support anti-inflammatory responses, membrane fluidity, and neurodevelopment. Olive oil, rich in omega-9, may improve lipid profiles and insulin sensitivity, and antioxidants from plant-based foods can reduce oxidative stress and modulate FA elongation and desaturation. Together, these mechanisms suggest that the MedDiet could play a key role in shaping lipid metabolism, cardiometabolic and neuropsychological health during this critical stage of development [43].

Although the differences in RBC FA levels between groups were statistically significant in our multivariate models (Table 3 and Table 4), the absolute changes reported in the descriptive Table 2 were modest (e.g., C20: 5 *n*-3: “poor-to-average adherence” 0.35% vs. “good adherence” 0.39%). The clinical and physiological relevance of such small variations in healthy adolescents remains uncertain. However, even subtle shifts in membrane FA composition may reflect longer-term dietary habits and could accumulate to exert metabolic effects over time, as suggested in both youth and adult populations. At cardiovascular level, a study assessed the Omega-3 Index in RBCs in a large sample of adolescents aged 13–15. Higher Omega-3 levels were associated with lower cardiovascular risk factors, including diastolic blood pressure and HDL cholesterol [44]. A study examined the association between RBC omega-3 fatty acids and cardiovascular risk in adults aged 30–74 without prior cardiovascular events. Higher RBC omega-3 levels were linked to lower odds of intermediate or high cardiovascular risk based on Framingham and Reynolds scores, suggesting that even modest changes in RBC fatty acid composition may have clinical relevance [45]. At mental health level. in a cross-sectional study using the WSS cohort, conducted in 372 adolescents (13.8 ± 0.9 years old), the RBC proportions of DHA and ALA were determined and found to be associated with attention scores [17]. In a cohort of postmenopausal women, lower RBC EPA + DHA levels correlated with smaller total and hippocampal brain volumes, the former being an indication of cognitive aging and the latter being centrally involved with Alzheimer’s disease pathology [46]. Therefore, despite our findings of only subtle differences between the MedDiet adherence groups and PUFAs in RBC, as part of our research question, these changes could still be associated at a physiological or clinical level. One key strength of this study lies in the analysis of lipidomics markers of diet, providing more precise information for the investigation of adherence to the MedDiet with RBCs FA composition. Using lipidomics techniques, we assessed 22 FAs in the RBC membrane, offering a more accurate long-term dietary indicator compared to total plasma or serum. This is because RBCs have a lifespan of approximately 120 days, allowing them to better reflect average dietary intake over time. From a metabolic perspective, FAs in RBC membranes exchange fatty acids with albumin, as well as high- and low-density lipoproteins. Since RBCs lack a nucleus, they continuously rely on FAs from dietary intake to maintain membrane homeostasis [15]. This reinforces the well-known metabolic relevance of measuring RBC FA composition as an indicator of physiological functioning during adolescence. Another strength of this study is the use of the KIDMED questionnaire, an adapted and validated instrument for the Spanish child and adolescent population [21]. Additionally, the use of PCA to create FA PC from highly correlated FAs enabled us to conduct confirmatory analyses based on the correlated FA approach. This method demonstrated statistical consistency with the individual associations observed between adherence to the MedDiet and FAs in the regression models. Finally, all analyzed associations were tested using multiple test comparison adjustments, with a maximum tolerance of 5% false positive results. This ensures the robustness of our findings [47].

Nevertheless, the current study also faced several limitations. The primary limitation is its cross-sectional design, which prevents the establishment of causality, as data were collected at a single time-point. The absence of longitudinal data also restricts the ability to comprehensively analyze the progression and dynamics of FAs and the MedDiet. Another limitation is the potential for selection bias, as the sample may not fully represent the broader population, potentially reflecting specific characteristics and inferences limited to the study sample. However, we used data from two different samples of two Spanish cities. Additionally, unmeasured environmental factors (e.g., environmental pollutants, family income) that influence both, adherence to the MedDiet and RBC FA composition may contribute to residual confounding making it challenging to account for the effects of omitted variables and thereby limiting the generalizability of the multivariate model results [48]. However, residual confounding is inherent to observational studies, and we included those confounders that we hypothesized may play the most important confounding role based on previous scientific literature. Nonetheless, the inclusion of two cohorts provided robust results regarding the association of MedDiet adherence with RBC FAs in adolescents. Unfortunately, using only overlapping data from WSS and INMA-Sabadell may have led to some loss of information regarding participants’ exposures, confounders, and outcomes in the study. Additionally, pooling baseline WSS data with 14-year INMA follow-up data may have introduced systematic bias due to differences in recruitment, socio-demographics, and measurement protocols. To account for this, cohort was included as a confounder and as an interaction term with MedDiet adherence in the analyses.

Future research should aim to provide further insight into the mechanisms that help explain the relationships between adherence to the MedDiet and saturated and polyunsaturated FA species in RBC as a reflection of dietary patterns in the adolescent population. This should be done taking into consideration a large amount of different fatty acids and adolescent samples from other countries and cultures with different dietary patterns. In addition, future longitudinal are warranted, potentially integrating FA biomarkers into long-term dietary surveillance systems to better monitor dieraty exposures in youth.

## 5. Conclusions

Overall, our findings suggest that a higher adherence to the MedDiet, as assessed by the KIDMED questionnaire, was directly associated with higher RBC EPA and DHA, as well as the PC primarily composed of omega-3 FAs (EPA, DHA, DPA, and ALA). In contrast, inverse associations were observed with AA and AdA. These association patterns are biological indicators of the potential influence of a healthy dietary pattern on adolescents during an important developing period.

## Figures and Tables

**Table 1:** Descriptive analysis of covariates from the WSS and INMA-Sabadell participants.

Characteristic	N	KIDMED		*p*-Value ^a^
		“Poor to Average Adherence” N = 428	“Good Adherence” N = 201	
KIDMED score	629	5.04 (1.69)	8.85 (0.97)	
Sex, *n* (%)	629			**0.006**
Male		203 (47%)	119 (59%)	
Female		225 (53%)	82 (41%)	
Age (years)	590	14.69 (1.10)	14.28 (1.22)	**<0.001**
Height (cm)	627	164.73 (8.30)	164.67 (9.59)	0.970
Weight (kg)	625	58.79 (13.36)	56.77 (13.69)	0.139
BMI z-score	586	0.39 (1.12)	0.30 (1.08)	0.340
Maternal education, *n* (%)	590			**<0.001**
No-university		217 (55%)	77 (40%)	
University		179 (45%)	117 (60%)	
Maternal social class	559			**0.035**
High and upper-middle		119 (31%)	77 (43%)	
Middle		192 (51%)	79 (44%)	
Working		67 (18%)	25 (14%)	
Cohort, *n* (%)	629			**<0.001**
INMA		247 (58%)	77 (38%)	
WSS		181 (42%)	124 (62%)	
Physical activity, *n* (%)	625			**<0.001**
Sedentary to low		120 (28%)	27 (14%)	
Moderate		110 (26%)	42 (21%)	
Active to quite active		196 (46%)	130 (65%)	

KIDMED score as adherence levels: “Poor-to-average adherence” (bellow or equal to 7) and “good adherence” (from equal 8 to 12). Fatty acids of red blood cell membranes are expressed as percentage (%). ^a^ Wilcoxon rank sum test was used for continuous variables and Pearson’s Chi-squared test was used for categorical variables.

**Table 2:** Descriptive analysis of fatty acids from the WSS and INMA-Sabadell participants (N = 629).

Characteristic	KIDMED		*p*-Value ^a^
	“Poor-to-Average Adherence” N = 428	“Good Adherence” N = 201	
KIDMED score	5.04 (1.69)	8.85 (0.97)	
C14:0	0.99 (0.85)	1.17 (1.01)	**0.003**
C16:0	19.63 (1.29)	19.92 (1.19)	**0.004**
C16:1 *n*-7	0.34 (0.17)	0.29 (0.16)	**<0.001**
C18:0	18.38 (1.14)	17.96 (1.28)	**<0.001**
All-trans C18:1	0.66 (0.46)	0.86 (0.46)	**<0.001**
C18:1 *n*-9 cis	14.84 (1.70)	15.43 (1.79)	**<0.001**
C18:2 *n*-6 cis	12.39 (1.43)	12.39 (1.62)	0.904
C18:3 *n*-6	0.10 (0.07)	0.13 (0.08)	**<0.001**
C18:3 *n*-3	0.10 (0.05)	0.11 (0.08)	0.074
C20:0	0.21 (0.05)	0.21 (0.05)	**0.035**
C20:1 *n*-9	0.32 (0.07)	0.30 (0.07)	**0.002**
C20:2 *n*-6	0.35 (0.08)	0.32 (0.12)	**<0.001**
C20:3 *n*-6	1.71 (0.37)	1.75 (0.39)	0.175
C20:4 *n*-6	17.97 (2.15)	16.91 (2.26)	**<0.001**
C20:5 *n*-3	0.35 (0.16)	0.39 (0.17)	**0.004**
C22:0	0.27 (0.12)	0.31 (0.12)	**<0.001**
C22:4 *n*-6	3.48 (0.61)	3.27 (0.60)	**<0.001**
C22:5 *n*-6	0.62 (0.14)	0.60 (0.15)	0.163
C22:5 *n*-3	1.40 (0.23)	1.43 (0.25)	0.204
C22:6 *n*-3	3.93 (0.91)	4.19 (0.86)	**0.001**
C24:0	0.61 (0.24)	0.69 (0.29)	**0.001**
C24:1 *n*-9	0.56 (0.28)	0.67 (0.31)	**<0.001**

KIDMED score as adherence levels: “Poor-to-average adherence” (bellow or equal to 7) and “good adherence” (from equal 8 to 12). Fatty acids of red blood cell membranes are expressed as percentage (%). ^a^ Wilcoxon rank sum test was used for continuous variables and Pearson’s Chi-squared test was used for categorical variables.

**Table 3:** Multivariate linear regressions between KIDMED adherence group and fatty acids of red blood cell membranes.

Characteristic	N	Coefficient ^a^	95% CI ^a^	*p*-Value
C16:0				
Poor-to-average adherence	368	Ref.		
Good adherence	184	0.18	0.01, 0.35	**0.041**
All-trans C18:1				
Poor-to-average adherence	368	Ref.		
Good adherence	184	0.05	0.01, 0.09	**0.016**
C20:4 *n*-6				
Poor-to-average adherence	368	Ref.		
Good adherence	184	−0.19	−0.30, −0.08	**<0.001**
C20:5 *n*-3				
Poor-to-average adherence	368	Ref.		
Good adherence	184	0.34	0.17, 0.52	**<0.001**
C22:4 *n*-6				
Poor-to-average adherence	368	Ref.		
Good adherence	184	−0.28	−0.46, −0.11	**0.002**
C22:5 *n*-6				
Poor-to-average adherence	368	Ref.		
Good adherence	184	−0.22	−0.40, −0.04	**0.018**
C22:6 *n*-3				
Poor-to-average adherence	368	Ref.		
Good adherence	184	0.29	0.11, 0.46	**0.001**

CI confidence interval. Ref. Reference group. Exposure beta coefficients with *p*-values bellow 0.05 are shown. All regression analyses are presented in the Supplementary Table S2. ^a^ Beta coefficient (slope) and 95% CI (Confidence Interval) estimated using multiple linear regression models adjusted for sex, age, BMI z-score, physical activity (“sedentary to low”, “moderate” and “active to quite active”, and maternal education (“up to high school”, “university”), and cohort (WSS/ INMA-Sabadell). Mediterranean Diet “Poor to Average Adherence” level, from KIDMED score, is used as reference value.

**Table 4:** Multivariate linear regression models between KIDMED adherence group and the principal components of fatty acid.

Characteristic	N	Coefficient ^a^	95% CI ^a^	*p*-Value
PC1 very-long chain FAs				
Poor-to-average adherence	368	Ref.		
Good adherence	184	0.04	−0.06, 0.14	0.442
PC2 long-chain omega-6 FAs				
Poor-to-average adherence	368	Ref.		
Good adherence	184	−0.19	−0.36, −0.03	**0.020**
PC3 omega-3 FAs				
Poor-to-average adherence	368	Ref.		
Good adherence	184	0.32	0.14, 0.49	**<0.001**

CI confidence interval, PC principal component, FA fatty acid. Principal component analysis was used to derive fatty acid patterns, retaining components with eigenvalues > 2.00. Three PCs were retained, accounting for 57.20% of the variance: PC1 very-long-chain FAs, PC2 long-chain omega-6 FAs, and PC3 omega 3 FAs. After varimax rotation, standardized scores were computed for each fatty acid PC. These standardized scores of PC were analyzed as outcomes in multiple linear regression. ^a^ Beta coefficient (slope) and 95% CI (Confidence Interval) estimated using multiple linear regression models adjusted for sex, age, BMI z-score, physical activity (“sedentary to low”, “moderate” and “active to quite active”, and maternal education (“up to high school”, “university”), cohort (WSS/ INMA-Sabadell). Mediterranean Diet “Poor to average adherence” level, from KIDMED score, is used as reference value. Ref. Reference group.

## Data Availability

Data described in the manuscript, code book, and analytic code will be made available upon request. Further inquiries can be directed to the corresponding author.

## Supplementary Materials

The following supporting information can be downloaded at: https://www.mdpi.com/article/10.3390/nu17172888/s1, Figure S1: Flowchart of participants. WSS = Walnuts Smart Snack Intervention Trial; INMA = Childhood and Environment, “INfancia y Medio Ambiente”; Figure S2: Density plot of fatty acids from WSS and INMA-Sabadell cohorts; Figure S3: Correlation plot between fatty acids and continuous KIDMED score. Correlations correspond to Spearman’s correlation. Only *p*-values below 0.05 are shown; Figure S4: Scree plot of principal component analysis in fatty acids of red blood cell membrane; Figure S5: Biplot for fatty acids of principal component 1, 2 and 3. PC1 very-long chain FAs; PC2 long-chain omega-6 FAs; PC3 omega-3 FAs; Table S1: Multivariate linear regressions between KIDMED adherence group and fatty acids of red blood cell membranes; Table S2: Principal component, eigenvalues, and cumulative variance in principal components analysis; Table S3: Spearman correlation between fatty acid (%) and standardized scores of principal components. Table S4: Multivariate regression models (Table 2 and Table 3) corrected p-values for multiple testing using the Benjamini-Hochberg false discovery rate. Table S5: Stratification by sex: Multivariate linear regressions between KIDMED adherence group and fatty acids of red blood cell membranes. Table S6: Sensitivity analysis by body mass index: multivariate linear regressions between KIDMED adherence groups and fatty acids in red blood cell membranes. Table S7: Sensitivity analysis by maternal social class: multivariate linear regressions between KIDMED adherence groups and fatty acids in red blood cell membranes. Table S8: Interaction analysis by cohort: multivariate linear regressions between KIDMED adherence groups and fatty acids in red blood cell membranes.

## Abbreviations

AdA	Adrenic acid
ALA	Alpha-linolenic acid
BMI	Body mass index
DHA	Docosahexaenoic acid
DPA	Docosapentaenoic acid
DPA omega-6	Docosapentaenoic omega-6
EPA	Eicosapentaenoic acid
FA	Fatty acids
FDR	False discovery rate
GLA	Gamma-linolenic acid
INMA	Spanish birth cohort of the Childhood and Environment
LA	Linoleic acid
MedDiet	Mediterranean diet
PC	Principal component
PCA	Principal component analysis
PUFAs	Polyunsaturated fatty acids
RBC	Red blood cells
SFA	Saturated fatty acid
WSS	Walnuts Smart Snack Dietary Intervention Trial
