# Supplementary material for: The Association Between the Mediterranean Diet and Fatty Acids in Red Blood Cells of Spanish Adolescents
Source: Nutrients. 2025 Sep 6;17(17):2888. doi: 10.3390/nu17172888 (PMC12430225; doi:10.3390/nu17172888)
Supplement: Supplementary file 1 [file nutrients-17-02888-s001.zip › nutrients-3831722-supplementary.pdf]

Supplementary Table S1. Multivariate linear regressions between KIDMED adherence group and fatty acids of red blood cell membranes.

Characteristic	N	Coefficient [a]	95% CI [a]	p-value
C14:0				
Low to moderate adherence	368	Ref.		
High adherence	184	0.03	-0.12, 0.19	0.665
C16:0				
Low to moderate adherence	368	Ref.		
High adherence	184	0.18	0.01, 0.35	**0.041**
C16:1 n-7				
Low to moderate adherence	368	Ref.		
High adherence	184	-0.06	-0.21, 0.09	0.420
C18:0				
Low to moderate adherence	368	Ref.		
High adherence	184	-0.04	-0.17, 0.08	0.501
All-trans C18:1				
Low to moderate adherence	368	Ref.		
High adherence	184	0.05	0.01, 0.09	**0.016**
C18:1 n-9 cis				
Low to moderate adherence	368	Ref.		
High adherence	184	0.08	-0.06, 0.22	0.267
C18:2 n-6 cis				
Low to moderate adherence	368	Ref.		
High adherence	184	-0.09	-0.26, 0.08	0.301
C18:3 n-6				
Low to moderate adherence	368	Ref.		
High adherence	184	0.01	-0.13, 0.16	0.843
C18:3 n-3				
Low to moderate adherence	368	Ref.		
High adherence	184	0.10	-0.09, 0.30	0.291
C20:0				
Low to moderate adherence	368	Ref.		
High adherence	184	-0.12	-0.27, 0.04	0.133
C20:1 n-9				
Low to moderate adherence	368	Ref.		
High adherence	184	-0.01	-0.16, 0.14	0.885
C20:2 n-6				
Low to moderate adherence	368	Ref.		
High adherence	184	-0.11	-0.28, 0.07	0.235
C20:3 n-6				
Low to moderate adherence	368	Ref.		
High adherence	184	-0.05	-0.23, 0.13	0.618
C20:4 n-6				
Low to moderate adherence	368	Ref.		
High adherence	184	-0.19	-0.30, -0.08	**<0.001**
C20:5 n-3				
Low to moderate adherence	368	Ref.		
High adherence	184	0.34	0.17, 0.52	**<0.001**
C22:0				
Low to moderate adherence	368			
High adherence	184	-0.03	-0.14, 0.07	0.521
C22:4 n-6				
Low to moderate adherence	368	Ref.		
High adherence	184	-0.28	-0.46, -0.11	**0.002**
C22:5 n-6				
Low to moderate adherence	368	Ref.		
High adherence	184	-0.22	-0.40, -0.04	**0.018**
C22:5 n-3				
Low to moderate adherence	368	Ref.	Ref.	
High adherence	184	0.13	-0.04, 0.30	0.135
C22:6 n-3				
Low to moderate adherence	368	Ref.		
High adherence	184	0.29	0.11, 0.46	**0.001**
C24:0				
Low to moderate adherence	368	Ref.	Ref.	
High adherence	184	-0.01	-0.11, 0.10	0.887
C24:1 n-9				
Low to moderate adherence	368	—	—	
High adherence	184	0.07	-0.03, 0.16	0.156

CI Confidence Interval, Ref. reference group. [a] Beta coefficient (slope) and 95% CI (Confidence Interval) estimated using multiple linear regression models adjusted for sex, age, BMI z-score, physical activity(“Sedentary to low”, “moderate” and “active to quite active”, maternal education (“No-university” , “university”), and cohort (WSS/ INMA-Sabadell). KIDMED adherence “Low to moderate adherence” level is used as reference value.

Supplementary Table S2. Principal component, eigenvalues, and cumulative variance in principal components analysis.

PC	Eigenvalue	Variance (%)	Cumulative variance (%)
1	7.42	33.75	33.75
2	2.91	13.22	46.98
3	2.24	10.21	57.20
4	1.81	8.23	65.43
5	1.39	6.32	71.76
6	1.01	4.59	76.36
…	…	…	…
22	0.00	0.02	100.00

Supplementary Table S3. Spearman correlation between fatty acid (%) and standardized scores of principal components.

FA from RBCs (%)	PC1 very-long chain FAs	PC2 long-chain omega-6 FAs	PC3 omega-3 FAs
C16:1 n-7	-0.69*	0.07	0.08*
C18:0	-0.66*	0.51*	-0.07
All-trans C18:1	0.74*	-0.39*	0.06
C18:1 n-9 cis	0.34*	-0.70*	-0.03
C18:2 n-6 cis	0.39*	-0.08	-0.17*
C18:3 n-6	0.76*	-0.36*	-0.09*
C18:3 n-3	-0.03	-0.23*	0.24*
C20:0	0.63*	-0.22*	0.02
C20:1 n-9	-0.42*	0.47*	0.10*
C20:2 n-6	-0.25*	0.67*	-0.08
C20:3 n-6	0.52*	0.29*	-0.03
C20:4 n-6	-0.61*	0.65*	-0.04
C20:5 n-3	-0.25*	0.02	0.87*
C22:0	0.91*	-0.17*	0.02
C22:4 n-6	-0.01	0.68*	-0.48*
C22:5 n-6	0.41*	0.50*	-0.40*
C22:5 n-3	-0.06	0.24*	0.69*
C22:6 n-3	0.01	-0.06	0.85*
C24:0	0.90*	-0.17*	0.00
C24:1 n-9	0.90*	-0.15*	0.09*

PC principal component, FA fatty acids, RBCs red blood cells.

*p-value <0.05

Supplementary Table S4. Multivariate regression models (Table 2 and 3) corrected p-values for multiple testing using the Benjamini-Hochberg false discovery rate.

**Outcome**	**p value**	**rank**	**Benjamini-Hochberg critical (q-value)**
C20:5 n-3	<0.001	1	0.002
C20:4 n-6	<0.001	2	0.004
PC3 omega-3	<0.001	3	0.006
C22:6 n-3	0.001	4	0.008
C22:4 n-6	0.002	5	0.010
All-trans C18:1	0.016	6	0.012
C22:5 n-6	0.018	7	0.014
PC2 long-chain omega 6	0.020	8	0.016
C16:0	0.041	9	0.018
C20:0	0.133	10	0.020
C22:5 n-3	0.135	11	0.022
C24:1 n-9	0.156	12	0.024
C20:2 n-6	0.235	13	0.026
C18:1 n-9 cis	0.267	14	0.028
C18:3 n-3	0.291	15	0.030
C18:2 n-6 cis	0.301	16	0.032
C16:1 n-7	0.420	17	0.034
PC1 very-long chain	0.442	18	0.036
C18:0	0.501	19	0.038
C22:0	0.521	20	0.040
C20:3 n-6	0.618	21	0.042
C14:0	0.665	22	0.044
C18:3 n-6	0.843	23	0.046
C20:1 n-9	0.885	24	0.048
C24:0	0.887	25	0.050

Only p-values smaller than the corresponding q-values were considered statistically significant and useful for statistical inference.

Supplementary Table S5. Stratification by sex: Multivariate linear regressions between KIDMED adherence group and fatty acids of red blood cell membranes.

Characteristic	Exposure	**Model 1.** Sex = Female			**Model 2.** Sex= Male		
		**Coefficient [a]**	**95% CI [a]**	**p value**	**Coefficient**	**95% CI**	**p value**
C14:0	Poor to average adherence	Ref.			Ref.		
	Good adherence	0.04	-0.22, 0.29	0.783	0.03	-0.17, 0.22	0.767
C16:0	Poor to average adherence	Ref.			Ref.		
	Good adherence	0.19	-0.06, 0.45	0.133	0.16	-0.07, 0.40	0.180
C16:1 n-7	Poor to average adherence	Ref.			Ref.		
	Good adherence	-0.14	-0.37, 0.09	0.234	-0.01	-0.20, 0.17	0.875
C18:0	Poor to average adherence	Ref.			Ref.		
	Good adherence	0.09	-0.10, 0.28	0.360	-0.14	-0.31, 0.02	0.093
All-trans C18:1	Poor to average adherence	Ref.			Ref.		
	Good adherence	0.04	-0.02, 0.10	0.223	0.06	0.00, 0.12	**0.036**
C18:1 n-9 cis	Poor to average adherence	Ref.			Ref.	Ref	
	Good adherence	0.06	-0.15, 0.27	0.571	0.08	-0.11, 0.28	0.408
C18:2 n-6 cis	Poor to average adherence	Ref.			Ref.		
	Good adherence	0.08	-0.18, 0.34	0.538	-0.22	-0.45, 0.01	0.062
C18:3 n-6	Poor to average adherence	Ref.			Ref.		
	Good adherence	-0.01	-0.24, 0.21	0.900	0.03	-0.15, 0.22	0.726
C18:3 n-3	Poor to average adherence	Ref.			Ref.		
	Good adherence	0.04	-0.20, 0.28	0.765	0.15	-0.15, 0.45	0.327
C20:0	Poor to average adherence	Ref.			Ref.	Ref	
	Good adherence	-0.11	-0.34, 0.12	0.336	-0.13	-0.34, 0.08	0.222
C20:1 n-9	Poor to average adherence	Ref.			Ref.	Ref	
	Good adherence	-0.03	-0.25, 0.19	0.764	-0.01	-0.22, 0.21	0.955
C20:2 n-6	Poor to average adherence	Ref.			Ref.		
	Good adherence	-0.03	-0.20, 0.15	0.763	-0.17	-0.47, 0.13	0.270
C20:3 n-6	Poor to average adherence	Ref.			Ref		
	Good adherence	-0.13	-0.38, 0.12	0.314	0.02	-0.23, 0.28	0.861
C20:4 n-6	Poor to average adherence	Ref.			Ref.	Ref	
	Good adherence	-0.25	-0.42, -0.07	0.005	-0.13	-0.27, 0.01	0.074
C20:5 n-3	Poor to average adherence	Ref.			Ref.		
	Good adherence	0.19	-0.09, 0.47	0.183	0.46	0.24, 0.68	**<0.001**
C22:0	Poor to average adherence	Ref.			Ref.		
	Good adherence	-0.11	-0.29, 0.07	0.232	0.04	-0.07, 0.15	0.518
C22:4 n-6	Poor to average adherence	Ref.			Ref.		
	Good adherence	-0.34	-0.61, -0.08	**0.012**	-0.22	-0.46, 0.01	0.065
C22:5 n-6	Poor to average adherence	Ref.			Ref.		
	Good adherence	-0.37	-0.63, -0.12	**0.005**	-0.09	-0.35, 0.16	0.470
C22:5 n-3	Poor to average adherence	Ref.			Ref.		
	Good adherence	-0.02	-0.29, 0.24	0.851	0.26	0.02, 0.49	**0.030**
C22:6 n-3	Poor to average adherence	Ref.					
	Good adherence	0.24	-0.04, 0.52	0.093	0.32	0.09, 0.54	**0.006**
C24:0	Poor to average adherence	Ref.			Ref.		
	Good adherence	-0.03	-0.17, 0.11	0.683	0.02	-0.14, 0.18	0.791
PC1 very-long chain FAs	Poor to average adherence	Ref.			Ref.		
	Good adherence	-0.10	-0.24, 0.05	0.201	0.01	-0.12, 0.15	0.834
PC2 long-chain omega-6 FAs	Poor to average adherence	Ref.			Ref.		
	Good adherence	-0.25	-0.49, -0.01	**0.045**	-0.14	-0.36, 0.08	0.215
PC3 omega-3 FAs	Poor to average adherence	Ref.			Ref.		
	Good adherence	0.19	-0.08, 0.47	0.170	0.41	0.18, 0.64	**<0.001**

CI Confidence Interval, Ref. reference group. [a] Beta coefficient (slope) and 95% CI (Confidence Interval) estimated using multiple linear regression models adjusted for age, BMI z-score, physical activity(“Sedentary to low”, “moderate” and “active to quite active”, maternal education (“No-university” , “university”), and cohort (WSS/ INMA-Sabadell). KIDMED adherence “Poor to average adherence” level is used as reference value. Model 1 represents the female dataset (n = 198 “Poor to average adherence”, n = 75 “Good adherence”), while Model 2 represents the male dataset (n = 170 “Poor to average adherence”, n = 109 “Good adherence”).

Supplementary Table S6. Sensitivity analysis by body mass index: multivariate linear regressions between KIDMED adherence groups and fatty acids in red blood cell membranes.

**Characteristic**	**Exposure**	**Model 1.**			**Model 2.** Model 1 + BMI z-score		
		**Coefficient [a]**	**95% CI [a]**	**p-value**	**Coefficient**	**95% CI**	**p-value**
C14:0	Poor to average adherence				Ref.		
	Good adherence	0.03	-0.12, 0.19	0.684	0.03	-0.12, 0.19	0.665
C16:0	Poor to average adherence	Ref.			Ref.		
	Good adherence	0.18	0.01, 0.35	**0.041**	0.18	0.01, 0.35	**0.041**
C16:1 n-7	Poor to average adherence	Ref.			Ref.		
	Good adherence	-0.06	-0.21, 0.08	0.400	-0.06	-0.21, 0.09	0.420
C18:0	Poor to average adherence	Ref.			Ref.		
	Good adherence	-0.04	-0.17, 0.08	0.517	-0.04	-0.17, 0.08	0.501
All-trans C18:1	Poor to average adherence	Ref.			Ref.		
	Good adherence	0.05	0.01, 0.09	**0.016**	0.05	0.01, 0.09	**0.016**
C18:1 n-9 cis	Poor to average adherence	Ref.			Ref.		
	Good adherence	0.08	-0.06, 0.23	0.258	0.08	-0.06, 0.22	0.267
C18:2 n-6 cis	Poor to average adherence	Ref.			Ref.		
	Good adherence	-0.09	-0.26, 0.08	0.308	-0.09	-0.26, 0.08	0.301
C18:3 n-6	Poor to average adherence	Ref.			Ref.		
	Good adherence	0.01	-0.13, 0.16	0.875	0.01	-0.13, 0.16	0.843
C18:3 n-3	Poor to average adherence	Ref.			Ref.		
	Good adherence	0.11	-0.09, 0.30	0.277	0.10	-0.09, 0.30	0.291
C20:0	Poor to average adherence	Ref.			Ref.		
	Good adherence	-0.11	-0.27, 0.04	0.141	-0.12	-0.27, 0.04	0.133
C20:1 n-9	Poor to average adherence	Ref.			Ref.		
	Good adherence	-0.01	-0.16, 0.14	0.907	-0.01	-0.16, 0.14	0.885
C20:2 n-6	Poor to average adherence	Ref.			Ref.		
	Good adherence	-0.11	-0.29, 0.07	0.223	-0.11	-0.28, 0.07	0.235
C20:3 n-6	Poor to average adherence	Ref.			Ref.		
	Good adherence	-0.05	-0.23, 0.13	0.582	-0.05	-0.23, 0.13	0.618
C20:4 n-6	Poor to average adherence	Ref.			Ref.		
	Good adherence	-0.19	-0.30, -0.08	**<0.001**	-0.19	-0.30, -0.08	**<0.001**
C20:5 n-3	Poor to average adherence	Ref.			Ref.		
	Good adherence	0.35	0.17, 0.52	**<0.001**	0.34	0.17, 0.52	**<0.001**
C22:0	Poor to average adherence	Ref.			Ref.		
	Good adherence	-0.03	-0.14, 0.07	0.529	-0.03	-0.14, 0.07	0.521
C22:4 n-6	Poor to average adherence	Ref.			Ref.		
	Good adherence	-0.29	-0.47, -0.11	**0.002**	-0.28	-0.46, -0.11	**0.002**
C22:5 n-6	Poor to average adherence	Ref.			Ref.		
	Good adherence	-0.22	-0.40, -0.04	**0.017**	-0.22	-0.40, -0.04	**0.018**
C22:5 n-3	Poor to average adherence	Ref.			Ref.		
	Good adherence	0.14	-0.04, 0.31	0.125	0.13	-0.04, 0.30	0.135
C22:6 n-3	Poor to average adherence	Ref.			Ref.		
	Good adherence	0.29	0.11, 0.47	**0.001**	0.29	0.11, 0.46	**0.001**
C24:0	Poor to average adherence	Ref.			Ref.		
	Good adherence	-0.01	-0.11, 0.10	0.892	-0.01	-0.11, 0.10	0.887
C24:1 n-9	Poor to average adherence	Ref.			Ref.		
	Good adherence	0.07	-0.03, 0.16	0.159	0.07	-0.03, 0.16	0.156
PC1 very-long chain FAs	Poor to average adherence	Ref.			Ref.		
	Good adherence	-0.04	-0.14, 0.06	0.437	-0.04	-0.14, 0.06	0.442
PC2 long-chain omega-6 FAs	Poor to average adherence	Ref.			Ref.		
	Good adherence	-0.20	-0.36, -0.03	**0.019**	-0.19	-0.36, -0.03	**0.020**
PC3 omega-3 FAs	Poor to average adherence	Ref.			Ref.		
	Good adherence	0.32	0.14, 0.50	**<0.001**	0.32	0.14, 0.49	**<0.001**

CI Confidence Interval, BMI body mass index, Ref. reference group; [a] Beta coefficient (slope) and 95% CI (Confidence Interval) estimated using multiple linear regression models. Model 1 adjusted for age, physical activity ("Sedentary to low", "moderate" and "active to quite active", maternal education ("No-university", "university"), and cohort (WSS/ INMA-Sabadell). Model 2 was adjusted for the variables in Model 1 and additionally for BMI z-score. The "Poor to average adherence" level of KIDMED adherence was used as the reference value. The sample size was equalized for both analyses ("Poor to average adherence" n = 368, "Good adherence" n = 184).

Supplementary Table S7. Sensitivity analysis by maternal social class: multivariate linear regressions between KIDMED adherence groups and fatty acids in red blood cell membranes.

**Outcome**	**Characteristic**	**Model 1.**			**Model 2.** Model 1 + maternal social class		
		**Coefficient [a]**	**95% CI [a]**	**p-value**	**Coefficient [a]**	**95% CI [a]**	**p-value**
C14:0	Poor to average adherence	Ref			Ref		
	Good adherence	0.02	-0.14, 0.18	0.815	0.02	-0.14, 0.18	0.787
C16:0	Poor to average adherence	Ref			Ref		
	Good adherence	0.20	0.01, 0.38	**0.036**	0.19	0.01, 0.38	**0.039**
C16:1 n-7	Poor to average adherence	Ref			Ref		
	Good adherence	-0.07	-0.22, 0.08	0.352	-0.07	-0.22, 0.08	0.370
C18:0	Poor to average adherence	Ref			Ref		
	Good adherence	-0.05	-0.18, 0.09	0.493	-0.05	-0.18, 0.09	0.500
All-trans C18:1	Poor to average adherence	Ref			Ref		
	Good adherence	0.06	0.01, 0.10	**0.013**	0.06	0.01, 0.10	**0.013**
C18:1 n-9 cis	Poor to average adherence	Ref			Ref		
	Good adherence	0.12	-0.02, 0.27	0.103	0.13	-0.02, 0.28	0.093
C18:2 n-6 cis	Poor to average adherence	Ref			Ref		
	Good adherence	-0.12	-0.30, 0.05	0.167	-0.13	-0.30, 0.05	0.157
C18:3 n-6	Poor to average adherence	Ref			Ref		
	Good adherence	0.00	-0.15, 0.15	0.994	0.00	-0.15, 0.15	0.982
C18:3 n-3	Poor to average adherence	Ref			Ref		
	Good adherence	0.12	-0.08, 0.32	0.247	0.12	-0.08, 0.32	0.251
C20:0	Poor to average adherence	Ref			Ref		
	Good adherence	-0.11	-0.27, 0.05	0.177	-0.11	-0.27, 0.05	0.185
C20:1 n-9	Poor to average adherence	Ref			Ref		
	Good adherence	-0.01	-0.17, 0.15	0.891	-0.01	-0.17, 0.15	0.888
C20:2 n-6	Poor to average adherence	Ref			Ref		
	Good adherence	-0.14	-0.33, 0.05	0.150	-0.14	-0.33, 0.05	0.150
C20:3 n-6	Poor to average adherence	Ref			Ref		
	Good adherence	-0.06	-0.25, 0.13	0.529	-0.06	-0.25, 0.13	0.542
C20:4 n-6	Poor to average adherence	Ref	Ref		Ref		
	Good adherence	-0.20	-0.31, -0.08	**<0.001**	-0.20	-0.31, -0.08	**<0.001**
C20:5 n-3	Poor to average adherence	Ref	Ref		Ref		
	Good adherence	0.32	0.13, 0.50	**<0.001**	0.32	0.13, 0.50	**<0.001**
C22:0	Poor to average adherence	Ref	Ref		Ref		
	Good adherence	-0.03	-0.14, 0.07	0.541	-0.04	-0.14, 0.07	0.506
C22:4 n-6	Poor to average adherence	Ref	Ref		Ref		
	Good adherence	-0.32	-0.50, -0.14	**<0.001**	-0.32	-0.50, -0.14	**<0.001**
C22:5 n-6	Poor to average adherence	Ref	Ref		Ref		
	Good adherence	-0.26	-0.44, -0.07	**0.006**	-0.26	-0.44, -0.08	**0.006**
C22:5 n-3	Poor to average adherence	Ref	Ref		Ref		
	Good adherence	0.17	-0.01, 0.35	0.070	0.17	-0.01, 0.35	0.060
C22:6 n-3	Poor to average adherence	Ref	Ref		Ref		
	Good adherence	0.31	0.13, 0.49	**<0.001**	0.31	0.12, 0.49	**0.001**
C24:0	Poor to average adherence	Ref	Ref		Ref		
	Good adherence	0.01	-0.10, 0.12	0.839	0.01	-0.10, 0.12	0.897
C24:1 n-9	Poor to average adherence	Ref	Ref		Ref		
	Good adherence	0.08	-0.01, 0.17	0.095	0.08	-0.02, 0.17	0.106
PC1 very-long chain FAs	Poor to average adherence	Ref	Ref		Ref		
	Good adherence	-0.04	-0.14, 0.06	0.418	-0.04	-0.14, 0.06	0.396
PC2 long-chain omega-6 FAs	Poor to average adherence	Ref	Ref		Ref		
	Good adherence	-0.22	-0.39, -0.05	**0.010**	-0.22	-0.39, -0.05	**0.010**
PC3 omega-3 FAs	Poor to average adherence	Ref	Ref		Ref		
	Good adherence	0.34	0.15, 0.53	**<0.001**	0.34	0.15, 0.53	**<0.001**

CI Confidence Interval, Ref. reference group. [a] Beta coefficient (slope) and 95% CI (Confidence Interval) estimated using multiple linear regression models. Model 1 adjusted for sex, age, physical activity (“Sedentary to low”, “moderate” and “active to quite active”, maternal education (“No-university”, “university”), and cohort (WSS/ INMA-Sabadell). Model 2 adjusted for variables in Model 1 and additionally for “maternal social class”. KIDMED adherence “Poor to average adherence” level is used as reference value. The sample size was equalized for both analyses ("Poor to average adherence" n = 368, "Good adherence" n = 184).

Supplementary Table S8. Interaction analysis by cohort: multivariate linear regressions between KIDMED adherence groups and fatty acids in red blood cell membranes.

Outcome	Exposure	Main effect			Interaction (KIDMED*cohort)		
		Coefficient [a]	95% CI [a]	p-value	Coefficient [a]	95% CI [a]	p-value
C140	Poor to average adherence	Ref					
	Good adherence	0.04	-0.22, 0.29	0.781	0.00	-0.32, 0.32	0.992
C160	Poor to average adherence	Ref					
	Good adherence	0.15	-0.12, 0.43	0.274	0.04	-0.31, 0.39	0.816
C161n7	Poor to average adherence	Ref					
	Good adherence	-0.08	-0.32, 0.15	0.482	0.04	-0.26, 0.34	0.798
C180	Poor to average adherence	Ref					
	Good adherence	0.00	-0.20, 0.20	0.996	-0.07	-0.33, 0.18	0.587
C181n9trans	Poor to average adherence	Ref					
	Good adherence	0.05	-0.02, 0.11	0.190	0.01	-0.08, 0.10	0.804
C181n9cis	Poor to average adherence	Ref					
	Good adherence	0.12	-0.11, 0.35	0.302	-0.06	-0.36, 0.23	0.664
C182n6cis	Poor to average adherence	Ref					
	Good adherence	-0.33	-0.60, -0.05	**0.020**	0.38	0.03, 0.73	**0.032**
C183n6	Poor to average adherence	Ref					
	Good adherence	-0.02	-0.25, 0.21	0.867	0.06	-0.24, 0.35	0.709
C183n3	Poor to average adherence	Ref					
	Good adherence	0.08	-0.23, 0.39	0.617	0.04	-0.35, 0.44	0.839
C200	Poor to average adherence	Ref					
	Good adherence	-0.23	-0.48, 0.01	0.059	0.19	-0.12, 0.50	0.223
C201n9	Poor to average adherence	Ref					
	Good adherence	-0.09	-0.33, 0.16	0.475	0.13	-0.18, 0.44	0.424
C202n6	Poor to average adherence	Ref					
	Good adherence	-0.28	-0.56, 0.01	0.056	0.28	-0.09, 0.64	0.134
C203n6	Poor to average adherence	Ref					
	Good adherence	-0.08	-0.37, 0.20	0.570	0.06	-0.30, 0.43	0.742
C204n6	Poor to average adherence	Ref					
	Good adherence	-0.16	-0.34, 0.02	0.074	-0.04	-0.27, 0.18	0.710
C205n3	Poor to average adherence	Ref					
	Good adherence	0.48	0.20, 0.76	**<0.001**	-0.23	-0.58, 0.13	0.214
C220	Poor to average adherence	Ref					
	Good adherence	-0.08	-0.24, 0.09	0.354	0.07	-0.14, 0.28	0.501
C224n6	Poor to average adherence	Ref					
	Good adherence	-0.43	-0.71, -0.15	**0.003**	0.24	-0.12, 0.60	0.190
C225n6	Poor to average adherence	Ref					
	Good adherence	-0.33	-0.62, -0.04	**0.025**	0.18	-0.19, 0.55	0.330
C225n3	Poor to average adherence	Ref					
	Good adherence	0.45	0.17, 0.72	**0.001**	-0.52	-0.86, -0.17	**0.004**
C226n3	Poor to average adherence	Ref					
	Good adherence	0.54	0.26, 0.82	**<0.001**	-0.42	-0.78, -0.06	**0.022**
C240	Poor to average adherence	Ref					
	Good adherence	-0.07	-0.24, 0.10	0.401	0.11	-0.11, 0.32	0.336
C241n9	Poor to average adherence	Ref					
	Good adherence	-0.01	-0.16, 0.14	0.882	0.13	-0.06, 0.32	0.185
PC1 very-long chain FAs	Poor to average adherence	Ref					
	Good adherence	-0.11	-0.27, 0.04	0.154	0.12	-0.08, 0.32	0.226
PC2 long-chain omega-6 FAs	Poor to average adherence	Ref					
	Good adherence	-0.26	-0.52, 0.00	0.051	0.11	-0.22, 0.44	0.522
PC3 omega-3 FAs	Poor to average adherence	Ref					
	Good adherence	0.57	0.29, 0.86	**<0.001**	-0.42	-0.78, -0.06	**0.021**

CI Confidence Interval, Ref reference group, WSS Walnuts Smart Snack Intervention Trial. [a] Beta coefficient (slope) and 95% CI (Confidence Interval) estimated using multiple linear regression models. The models were adjusted for sex, age, physical activity ("Sedentary to low", "moderate" and "active to quite active", maternal education ("No-university", "university"), cohort (WSS/ INMA-Sabadell) and KIDMED*cohort. KIDMED adherence "Poor to average adherence" level is used as reference value. The main effect shows the regression outputs of KIDMED ("Poor to average adherence," "Good adherence"), while the interaction shows the regression outputs of KIDMED * cohort (Good adherence * WSS) for the same model, respectively for each outcome.

Supplementary figure S1. Flowchart of participants.

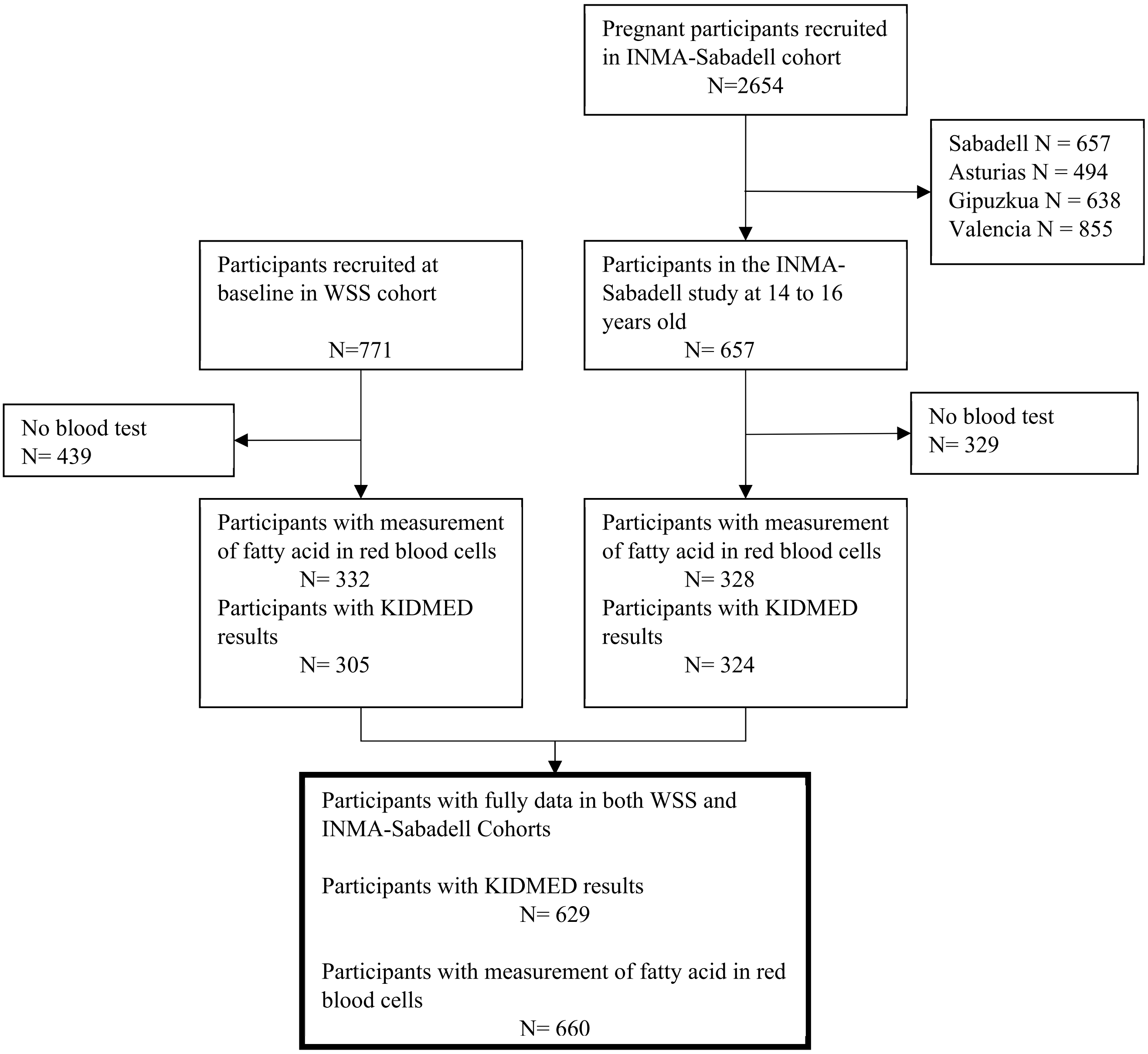

WSS= Walnuts Smart Snack Intervention Trial

INMA= Childhood and Environment, "INfancia y Medio Ambiente"

Supplementary figure S2. Density plot of fatty acids from WSS and INMA-Sabadell cohorts.

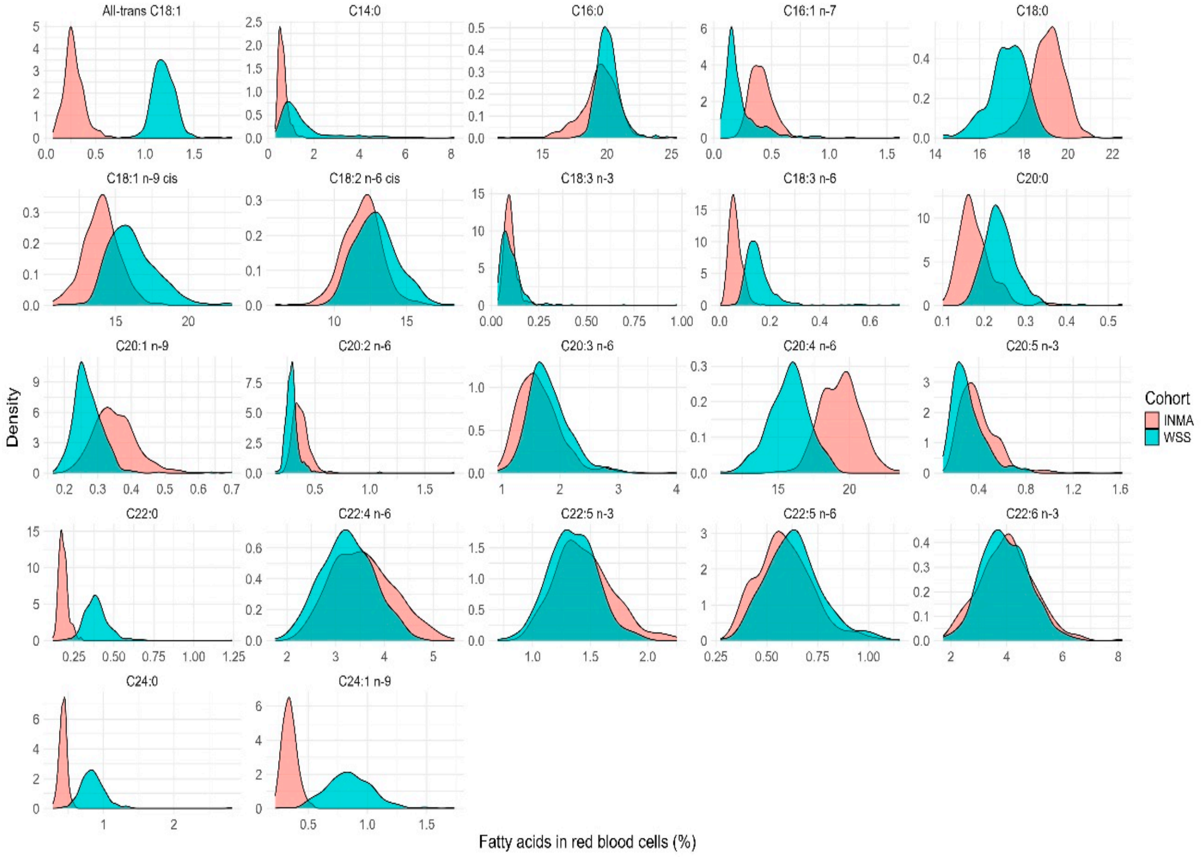

WSS= Walnuts Smart Snack Intervention Trial.

INMA= Childhood and Environment, “INfancia y Medio Ambiente”.

Supplementary figure S3. Correlation plot between fatty acids and continuous KIDMED score.

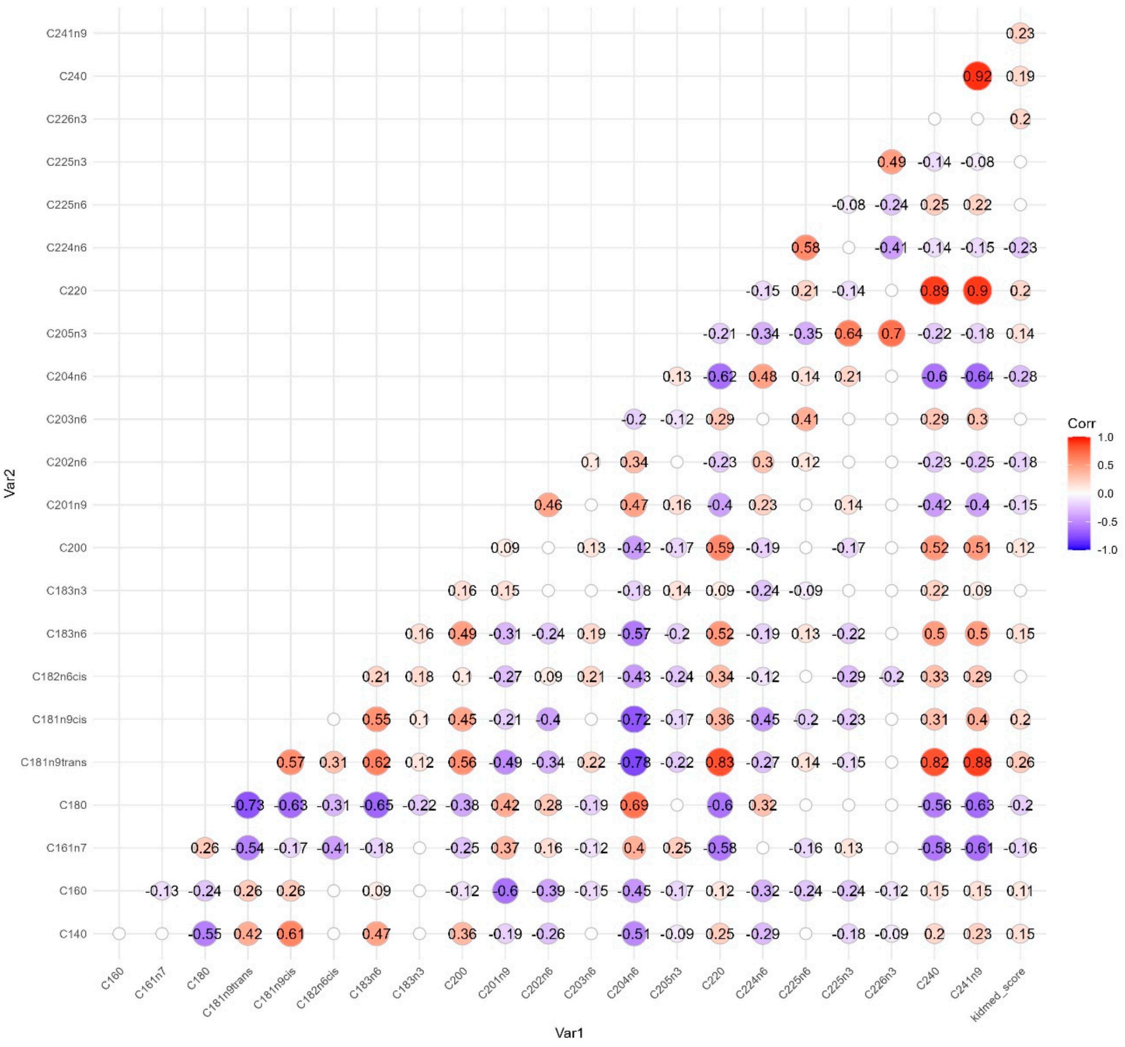

Correlations correspond to Spearman's correlation. Only p-values below 0.05 are shown

Supplementary figure S4. Scree plot of principal component analysis in fatty acids of red blood cell membrane.

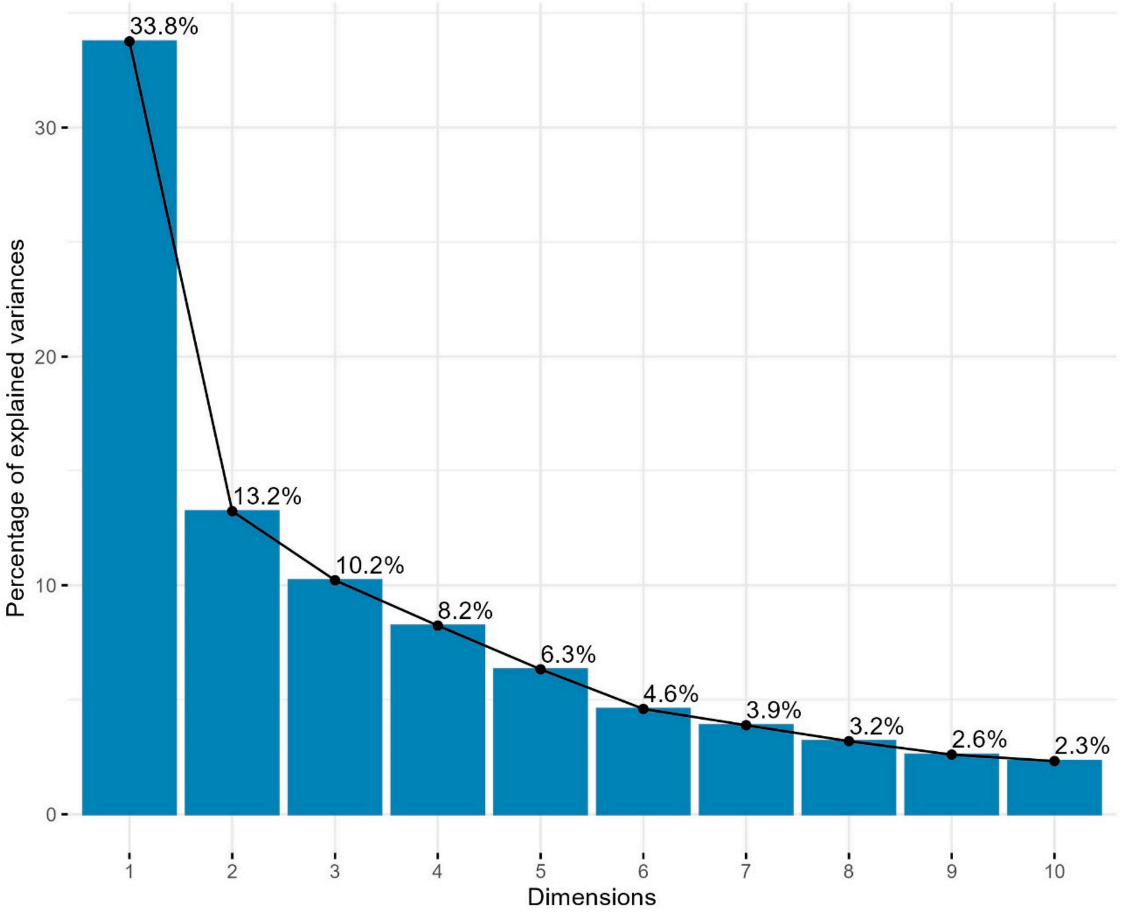

Supplementary figure S5. Biplot for fatty acids of principal component 1, 2 and 3.

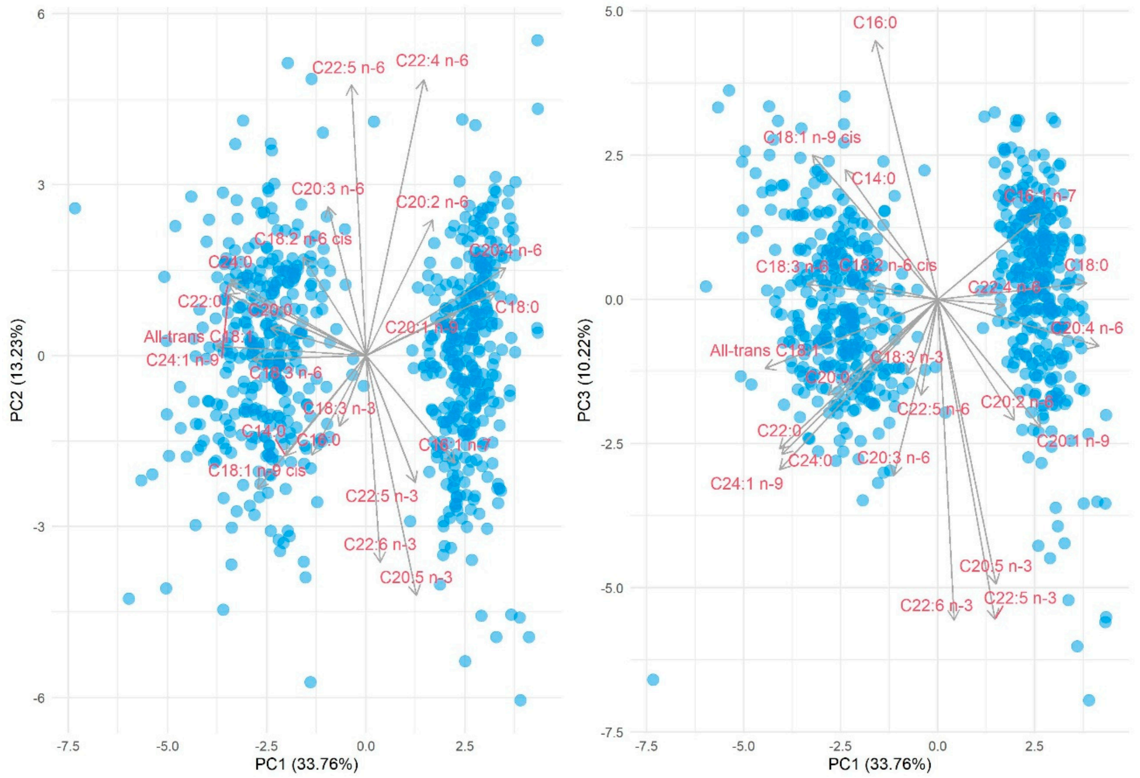

PC1 very-long chain FAs; PC2 long-chain omega-6 FAs; PC3 omega-3 FAs
